# Biliary parascarosis in a foal

**DOI:** 10.1002/vms3.1087

**Published:** 2023-02-01

**Authors:** Alireza Sazmand, Morteza Yavari, Mohammad Babaei, Alireza Nourian, Domenico Otranto

**Affiliations:** ^1^ Department of Pathobiology, Faculty of Veterinary Science Bu‐Ali Sina University Hamedan Iran; ^2^ Department of Clinical Sciences, Faculty of Veterinary Science Bu‐Ali Sina University Hamedan Iran; ^3^ Department of Veterinary Medicine University of Bari Aldo Moro Valenzano Bari Italy

**Keywords:** aberrant parasite migration, equine, *Nerium*, oleander, *Parascaris*

## Abstract

Migration of *Ascaris lumbricoides* through the papilla of Vater in humans, and entry into the biliary tree, is well‐recognised. *Ascaris suum* and *Toxocara vitulorum* have been recovered from the liver of swine and buffalo. We necropsied a Persian Kurdish filly at age 6 months, weighing ∼100 kg. Death evidently was caused by oleander (*Nerium oleander*) intoxication. An 8‐cm adult male *Parascaris* was found at the lobar‐left hepatic bile duct junction. We suggest that the nematode entered anteriorly into the hepatic tree, via the duodenum, major duodenal papilla, bile duct, left hepatic duct and finally the lobar duct. Considering the brief 4‐h elapsed time between death and necropsy, and the 18‐cm distance from the major duodenal papilla to the location of the parasite, we conclude that entry into the biliary tree likely occurred ante‐mortem. We advise consideration of *Parascaris* infection in differential diagnosis of equine hepatic and pancreatic dysfunction.

## Graphical abstract

Here we report finding of an adult male *Parascaris* nematode in the junction of the lobar duct and left hepatic duct during necropsy of a 6‐month‐old female foal who died because of oleander (*Nerium oleander*) intoxication.

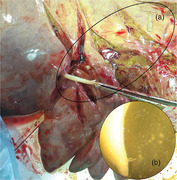

## 1 BACKGROUND

Infections by *Parascaris univalens* and *P. equorum* are common in horses (Nielsen, 2016), with high prevalence among foals aged less than one year (Scala et al., 2021). The life cycle of *Parascaris* is direct; no intermediate hosts are involved. Mature females occupy the small intestine, where they can shed hundreds of thousands of eggs each day. In the environment, the infective second stage larva (L2) is formed within the egg.

Following ingestion of the infective stage by the host, the released L2 larvae penetrate the small intestinal wall, beginning a somatic migration via the bloodstream through the liver, heart and lungs. From the respiratory system, mucosal secretions transport the larval stage upward to the larynx, to be swallowed. The 4th stage larvae arrive in the small intestine approximately 3 weeks post‐infection, resulting in a prepatent period of 10–16 weeks (Nielsen & Reinemeyer, 2018; Sazmand et al., 2020).

Small intestinal equine ascarids occasionally are recovered in low numbers from the stomach or cecum (Nielsen, 2016). Adult *Parascaris* in the equine liver or bile ducts have not been reported previously, insofar as the authors are aware. By contrast, there are many reports of *Ascaris lumbricoides* migration into the bile or pancreatic ducts of humans (John & Petri, 2006). Migration from the intestine through the ampulla of Vater into the biliary system of humans is a well‐known complication of *A. lumbricoides* infection (Khuroo & Zargar, 1985). In most cases, the worms return to the duodenum in 1–14 days (Das et al., 2007). However, complications such as biliary colic and obstructive jaundice have been reported, consequent to complete or partial obstruction of the bile duct, ascending and pyogenic cholangitis, cholecystolith formation, cholecystitis, intrahepatic abscess and pancreatitis (Fallah & Motahhari, 1998; Sandouk et al., 1997). Here we report, for the first time, an adult *Parascaris* in the lobar bile duct of a foal.

## 2 CASE PRESENTATION

In November 2020, a Persian Kurdish filly was examined in a suburb of Hamedan, Iran. The filly was aged about 6 months and weighed approximately 100 kg. The owner reported that the foal had been anorectic since the preceding day. The foal and mare had been fed with leaves from surrounding trees. The foal was lethargic and ataxic. Her abdomen was swollen, and she exhibited tachypnea and difficult expiration, without coughing. The owner had given the foal a single dose of 30 mL fenbendazole 2.5% oral suspension (7.5 mg/kg body weight, Rooyan Darou, Semnan, Iran) 6 days earlier, when *Parascaris* were observed in the faeces. During clinical examination, the foal developed systemic tremor, became recumbent, and died suddenly. Oleander (*Nerium oleander*) leaves were found in food remains, suggesting that death may have resulted from oleander intoxication.

The carcass was transferred to the Faculty of Veterinary Science, Bu‐Ali Sina Univesity  for further examination. At necropsy, the trachea contained frothy blood. The lungs were oedematous, with ecchymosis on the pleural surfaces. Hydropericardium was observed, along with scattered foci of pericardial and endocardial petechial haemorrhage. Foci of mild to severe haemorrhage were observed on digestive tract serosal surfaces. Petechial haemorrhages were noted on epithelial surfaces of the stomach and small intestine. The liver appeared oedematous and congested.

**FIGURE 1:**
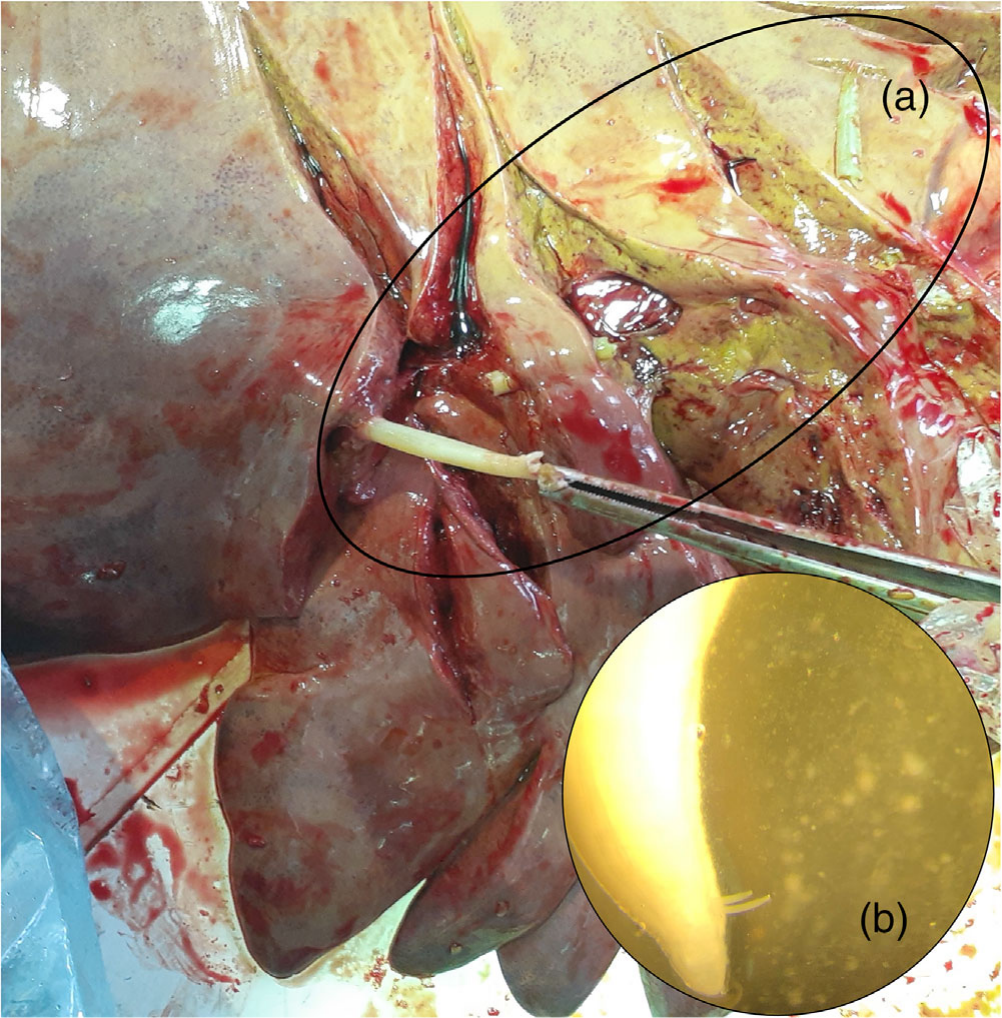
Adult *Parascaris* in the junction of the lobar duct and left hepatic duct. (a) Segments of the worm. (b) Spicules at posterior end showing that *Parascaris* was a male.

Oleander leaves were not found in the stomach. Approximately 100 adult *Parascaris* were found in the small intestine. Liver biliary ducts were examined thoroughly for *Fasciola* or other trematodes, which were not observed. An 8‐cm adult male *Parascaris* was found at the junction of the lobar and left hepatic ducts (Figure 1). Evidently, the parasite had entered the hepatic tree at its anterior end, and possible invasion pathway included duodenum, major duodenal papilla, bile duct, left hepatic duct and lobar duct. Considering the brief 4‐h interval between death and necropsy, and the 18 cm distance from the major duodenal papilla to the parasite's location, entry into the biliary tree very likely was ante‐mortem.

Sections of lung, liver and left ventricle of the heart were submitted for histopathological examination. The tissue specimens were processed routinely and stained with haematoxylin and eosin (H&E). Stained sections were examined with light microscopy and photographed with an attached digital camera.

**FIGURE 2:**
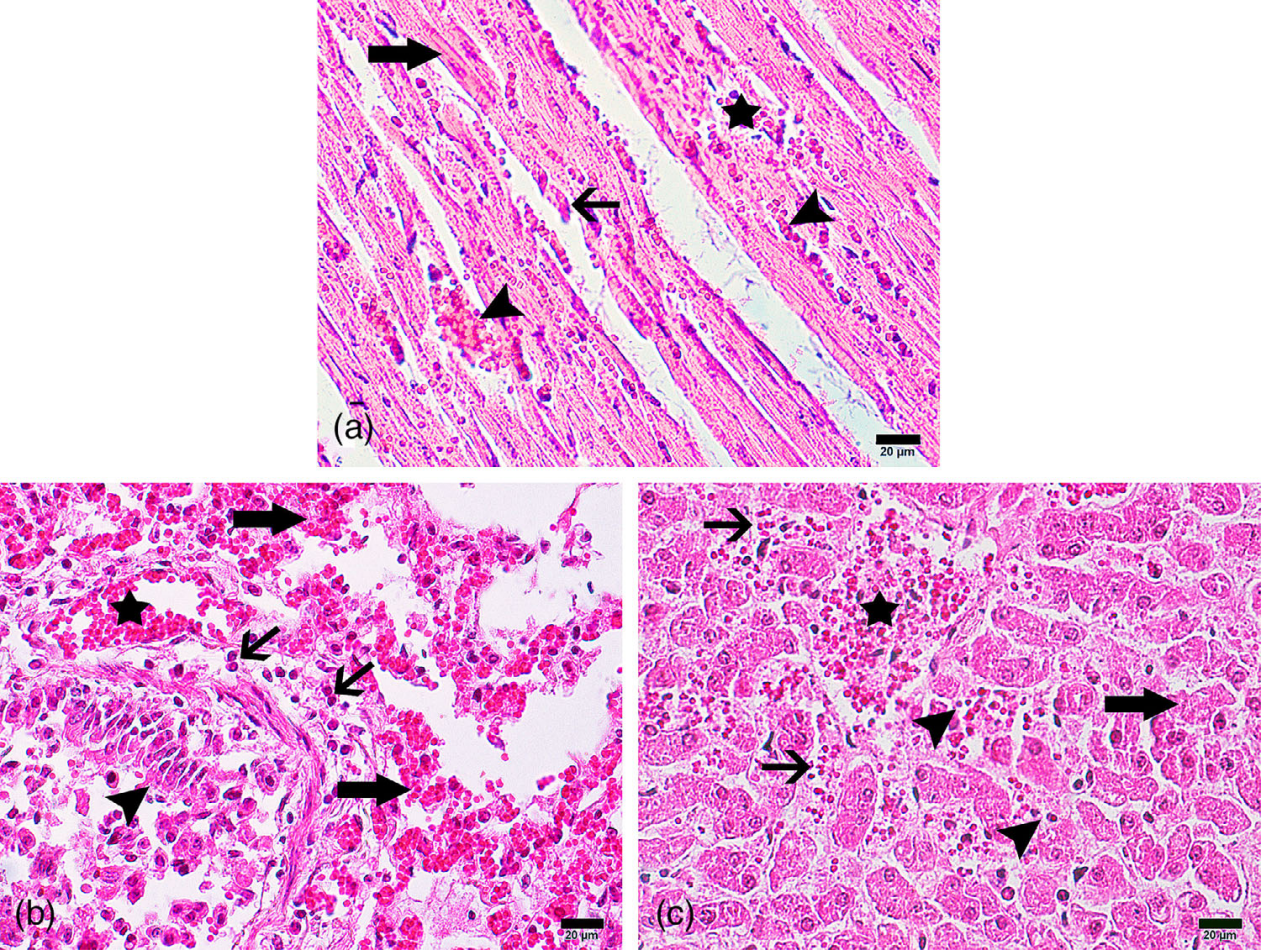
Light microscopic view of lesions in different organs following oleander intoxication. (a) Cardiac muscle shows myofibrillar necrosis (large arrow), sarcoplasmic fragmentation (small arrow), microvascular congestion (arrowheads) and interstitial oedema (asterisk). (b) Lung tissue demonstrates severe vascular congestion (asterisk) and interstitial haemorrhage (large arrows) along with leukocyte infiltration (small arrows) and interstitial oedema. Cellular necrosis and detachment of epithelium (arrowheads) is seen in bronchioles. (c) Liver shows hepatocellular necrosis (large arrow), central venous congestion (asterisk), oedema and distension of sinusoids (small arrows) along with mononuclear infiltration of leukocytes (arrowheads). Haematoxylin & Eosin, Magnification = 400×, Scale bar = 20 µm.

Cardiac lesions included severe multifocal necrosis and fragmentation of ventricular cardiomyocytes, microvascular congestion and interfibrillar oedema. Lung tissue revealed severe vascular congestion, interstitial haemorrhage and oedema, and an acute inflammatory (polymorphonuclear) infiltrate. Necrosis and detachment of bronchiolar respiratory epithelium were observed. Liver lesions included cellular degeneration, multifocal coagulative necrosis in acinar periportal and transitional zones, acute inflammation, venous congestion, oedema and distension of sinusoids and predominately mononuclear (lymphocytic) infiltrate (see Figure 2).

## 3 DISCUSSION

This first observation of adult *Parascaris* in the biliary tree of an equine host suggests parasite involvement in hepatopancreatic disease similar to human hepatobiliary and pancreatic duct ascariasis which is known to cause obstructive jaundice, pyogenic cholangitis, cholecystitis and pancreatitis (Fallah & Motahhari, 1998; Sandouk et al., 1997). Only a few reports have described migration of livestock ascarids into host biliary or pancreatic ducts [*Ascaris suum* in swine (Prchal, 1953) *Toxocara vitulorum* in buffalo (Srivastava & Sharma, 1981)]. We found just one previous report of *Parascaris* in an aberrant location in the horse, in the left ventricle of the heart (Pinto & Vaz, 1938).

The rarity of biliary parascarosis in equids could have an anatomical explanation. The horse common hepatic duct (∼5 cm long and 1–1.5 cm wide) is formed at the ventral aspect of the portal fissure by joining of the right and left hepatic ducts. The duct passes obliquely between the two layers of mesoduodenum, opens into the duodenum 12–15 cm from the pylorus, and empties into the hepatopancreatic ampulla. This anatomical arrangement forms a valve that prevents regurgitation of intestinal contents into the duct (Getty, 1975; Khalil et al., 2005).

In the present case, the *Parascaris* worm was removed from the lobar bile duct. No pathology of the biliary duct was found histologically, suggesting short‐term occupation. Migration of *A. lumbricoides* through the biliary tree, returning to the duodenum after a brief interval, does not cause clinical signs in humans. However, longer duct occupation can lead to complications such as biliary colic, cholecystolith formation, recurrent pyogenic cholangitis, cholecystitis, pancreatitis, hepatic abscess and septicaemia (Khuroo & Zargar, 1985; Das et al., 2007).

In swine, *A. suum* can migrate into the main bile ducts, gallbladder, hepatic bile ducts, pancreatic duct and pancreas. Longer duration of occupation has been linked directly to impairment of hepatic and pancreatic function (Prchal, 1953). In horses, however, only hepatic lesions have been reported in association with migrating *Parascaris* larvae (Brown & Clayton, 1979). Macroscopic observations included focal haemorrhages and small, white, diffuse or nodular lesions. Histological findings have included eosinophilic and lymphocytic infiltration, portal triad fibrosis, thrombosis and lymphoreticular nodules (Brown & Clayton, 1979). Elevated liver enzymes or clinical signs of liver disease have not been associated with equid parascarosis (Nielsen, 2016). However, biliary obstruction by *Parascaris* has been suggested as a consideration in differential diagnosis of liver disease (Milne, 1990).

The proximate cause of death in this report was oleander intoxication. *Nerium oleander* is an evergreen shrub that is cultivated worldwide as an ornamental plant. In ancient India, oleander was called Kajamaraka, ‘the herb that makes the horse die’. Oleander poisoning can be fatal for humans and animals; the lethal dose (LD) of dried leaves varies by species. Lethal doses have been reported by body weight in cattle (50 mg/kg), goats (100 mg/kg) and sheep (250 mg/kg) (Ceci et al., 2020; Aslani et al., 2007; Oryan et al., 1996). Equids are more susceptible to oleander poisoning than ruminants; a single dose of 30 mg/kg was fatal for donkeys in an experimental study (Rezakhani & Maham, 1994). Considering that fatal ingestion of oleander leaves can occur at 0.005% of an animal's body weight (Butler et al., 2016), the foal in the present report needed to consume just 0.5 kg of dried plant material.

It is believed that outmigration of *A. lumbricoides* from the intestinal lumen can be provoked by certain drugs and anaesthetic agents (John & Petri, 2006; Sanai & Al‐Karawi, 2007). Since oleander contains a variety of glycosides that include neriifolin, thevetin A and B and oleandrin (Bandara et al., 2010), aberrant migration of *Parascaris* in the present instance could have been associated with glycoside content of the host's ingested food. Whether the *Parascaris* nervous system is susceptible to oleander glycoside toxicity is not known, although flavonol glycosides of ssazainfoin (*Onobrychis viciifolia*) can inhibit migration of *Haemonchus contortus* larvae in vitro (Barrau et al., 2005). Additionally, cyanogenic glycosides of some plants have been associated with anti‐nematodal activity (Chitwood, 2002).

The recommended dose of fenbendazole is 7.5 mg/kg body weight, but resistance of gastrointestinal nematodes to anthelmintic compounds such as benzimidazoles is a common problem in Iran (Nemati et al., 2019). In particular, fenbendazole treatment failure was observed in three Iranian provinces namely Hamedan, Yazd and Azarbaiajan Sharghi (Ashrafzadeh et al., unpublished). Most regional farmers are not aware of this serious issue, and nonprescribed treatment with anthelmintics seems to be important for emergence of anthelmintic resistance (Sazmand et al., 2020). Hence, in the present case, the recent anthelmintic dose could have had a sublethal effect on the parasite.

Microscopic examination of heart, lung and liver revealed histopathology that is consistent with oleander intoxication in experimental studies (Aslani et al., 2007; Oryan et al., 1996; Khordadmehr et al., 2017) and naturally occurring oleander toxicosis in animals (Ceci et al., 2020; Hughes et al., 2002). Relevant findings have included myocardial necrosis and haemorrhage; effusion in pleural, peritoneal, and pericardial cavities; hepatocellular necrosis and hepatitis; gastrointestinal haemorrhage and oedema; pulmonary congestion; and renal infarction, necrosis, and congestion (Hughes et al., 2002; Renier et al., 2013; Bazargani et al., 2008). However, when sudden death occurs in oleander‐intoxicated animals, gross or microscopic lesions are observed rarely, due to insufficient elapsed time for development of an extensive inflammatory response (Aslani, 2018). The observed inflammatory foci in the liver parenchyma may have resulted from *Parascaris* larval migration in the organ rather than the effects of the ingested toxic plant.

## 4 CONCLUSION

Herein, the first observation of an adult *Parascaris* in the liver of a horse is presented. Although finding one adult without associated pathology does not allow casual conclusion it suggested that parascarosis should be considered in the differential diagnosis of foals and horses with hepatopancreatobiliary disease.

## AUTHOR CONTRIBUTIONS

Alireza Sazmand: Conceptualisation; investigation; methodology; project administration; supervision; writing – original draft. Morteza Yavari: Investigation. Mohammad Babaei: Investigation; Alireza Nourian: Investigation; writing – original draft. Domenico Otranto: Writing ‐ review & editing.

## FUNDING INFORMATION

The authors received no funding.

## CONFLICT OF INTEREST STATEMENT

The authors declare that they have no competing interests.

## ETHICS STATEMENT

Not applicable.

### PEER REVIEW

The peer review history for this article is available at https://publons.com/publon/10.1002/vms3.1087.

## Data Availability

All data generated or analysed during this study are included in this published article and its additional files.
